# Supplementary material for: Characterization of Mycoviruses in Armillaria ostoyae and A. cepistipes in the Czech Republic
Source: Viruses. 2024 Apr 15;16(4):610. doi: 10.3390/v16040610 (PMC11053624; doi:10.3390/v16040610)
Supplement: Supplementary file 1 [file viruses-16-00610-s001.zip › viruses-2946635-supplementary.pdf]

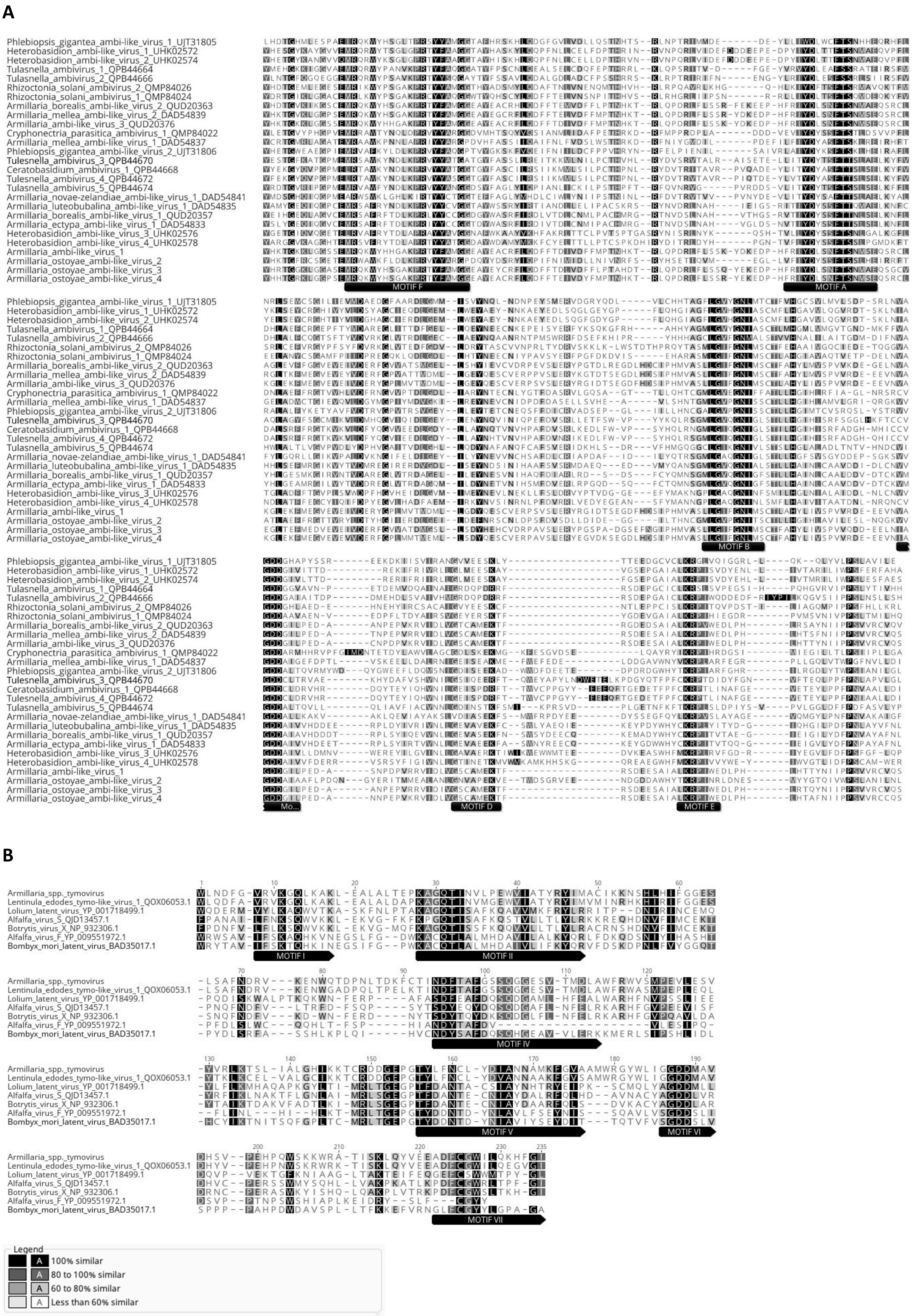
A
Phlebiopsis_gigantea_ambi-like_virus_1_UJT31805
Heterobasidion_ambi-like_virus_1_UHK02572
Heterobasidion_ambi-like_virus_2_UHK02574
Tulasnella_ambivirus_1_QPB44664
Tulasnella_ambivirus_2_QPB44666
Rhizoctonia_solani_ambivirus_2_QMP84026
Rhizoctonia_solani_ambivirus_1_QMP84024
Armillaria_borealis_ambi-like_virus_2_QUD20363
Armillaria_mellea_ambi-like_virus_2_DAD54839
Armillaria_ambi-like_virus_3_QUD20376
Cryphonectria_parasitica_ambivirus_1_QMP84022
Armillaria_mellea_ambi-like_virus_1_DAD54837
Phlebiopsis_gigantea_ambi-like_virus_2_UJT31806
Tulesnella_ambivirus_3_QPB44670
Ceratobasidium_ambivirus_1_QPB44668
Tulesnella_ambivirus_4_QPB44672
Tulesnella_ambivirus_5_QPB44674
Armillaria_novae-zelandiae_ambi-like_virus_1_DAD54841
Armillaria_luteobubalina_ambi-like_virus_1_DAD54835
Armillaria_borealis_ambi-like_virus_1_QUD20357
Armillaria_ectypa_ambi-like_virus_1_DAD54833
Heterobasidion_ambi-like_virus_3_UHK02576
Heterobasidion_ambi-like_virus_4_UHK02578
Armillaria_ambi-like_virus_1
Armillaria_ostoyae_ambi-like_virus_2
Armillaria_ostoyae_ambi-like_virus_3
Armillaria_ostoyae_ambi-like_virus_4
MOTIF F
MOTIF A
MOTIF B
Mo...
MOTIF D
MOTIF E
B
Armillaria_spp._tymovirus
Lentinula_edodes_tymo-like_virus_1_QOX06053.1
Lolium_latent_virus_YP_001718499.1
Alfalfa_virus_S_QJD13457.1
Botrytis_virus_X_NP_932306.1
Alfalfa_virus_F_YP_009551972.1
Bombyx_mori_latent_virus_BAD35017.1
MOTIF I
MOTIF II
MOTIF IV
MOTIF V
MOTIF VI
MOTIF VII
Legend
100% similar
80 to 100% similar
60 to 80% similar
Less than 60% similar

Figure S1. Amino acid alignment showing conserved motifs. **A,** Amino acid alignment of ambi-like viruses described in this study and related members. QPB44666 – Tulesnella ambivirus 2, QPB44664 – Tulesnella ambivirus 1, QMP84026 – Rhizoctonia solani ambivirus 2, QMP84024 – Rhizoctonia solani ambivirus 1, QUD20363 – Armillaria borealis ambi-like virus 2, AoALV3 – Armillaria ostoyae ambi-like virus 3*, DAD54839 – Armillaria mellea ambi-like virus 2, AoALV4 - Armillaria ostoyae ambi-like virus 4*, AoALV1 - Armillaria ambi-like virus 1*, QUD20376 – Armillaria ambi-like virus 3, QMP84022 – Cryphonectria parasitica ambivirus 1, AoALV2 - Armillaria ostoyae ambi-like virus 2*, DAD54837 – Armillaria mellea ambi-like virus 1, QPB44674 – Tulesnella ambivirus 5, DAD54841 – Armillaria novae-zelandiae ambi-like virus 1, DAD54835 – Armillaria luteobubalina ambi-like virus 1, QUD20357 – Armillaria borealis ambi-like virus 1, DAD54833 – Armillaria ectypa ambi-like virus 1, QPB44670 – Tulesnella ambivirus 3, QPB44672 – Tulesnella ambivirus 4, QPB44668 – Ceratobasidium ambivirus 1
B, Amino acid aligment of Armillaria tymovirus described in this study and related members. AoTV – Armillaria spp. tymovirus*, QOX06053.1 - Lentinula edodes tymo-like virus 1, YP_001718499.1 - Lolium latent virus, QJD13457.1 - Alfalfa virus S, NP_932306.1 - Botrytis virus X, BAD35017.1 - Bombyx mori latent virus, YP_009551972.1 - Alfalfa virus F.

(A)

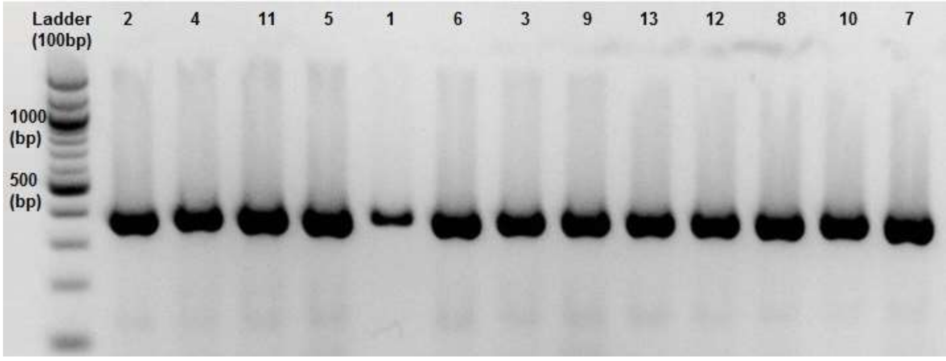

(B)

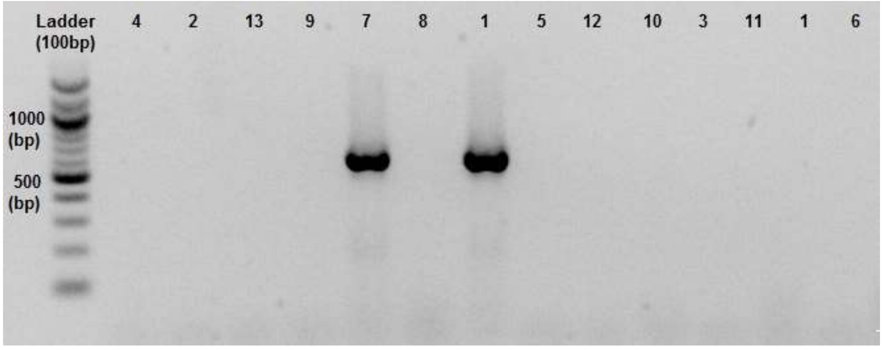

(C)

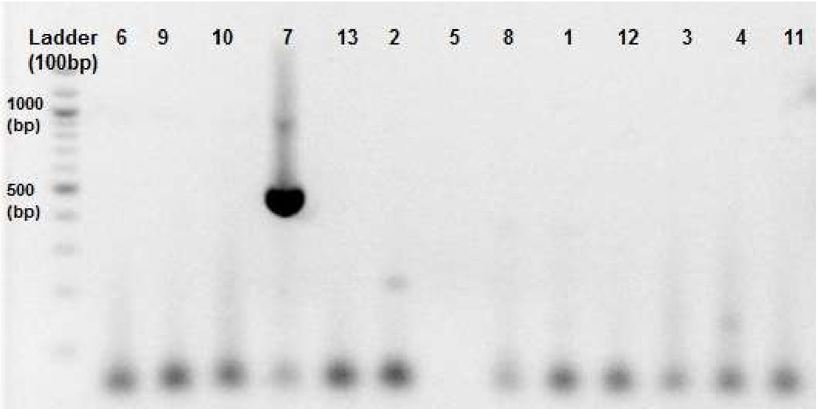

(D)

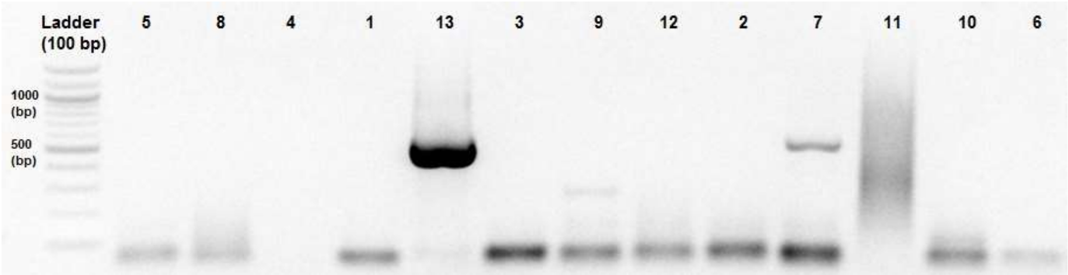

(E)

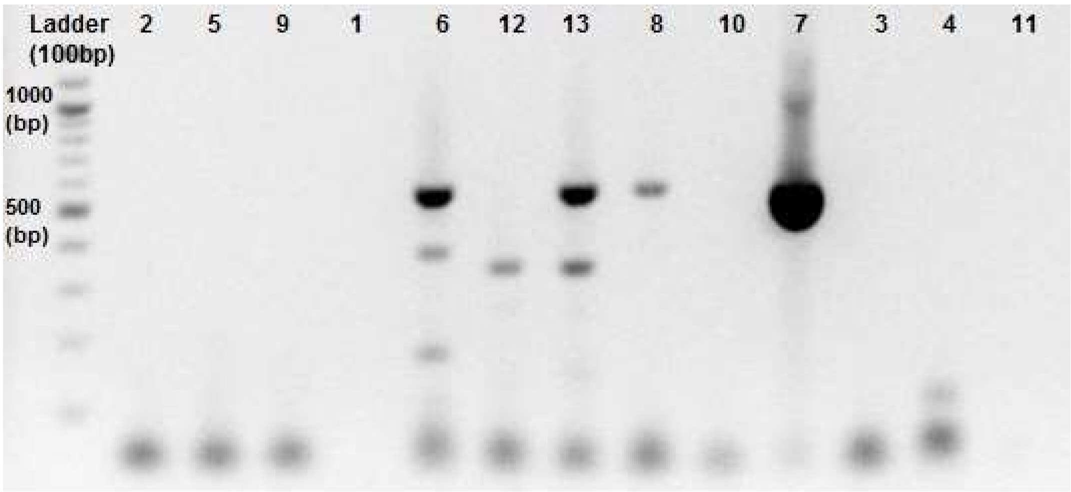

(F)

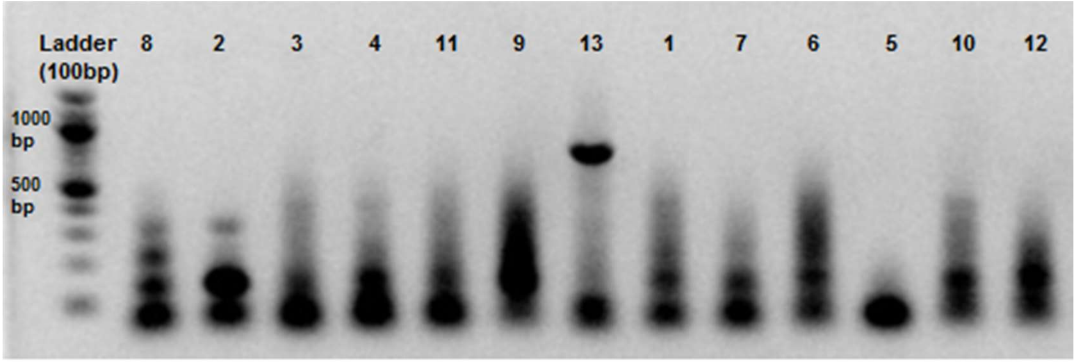

Figure S2. Resulting RT-PCR gels. **A,** Positive control with EF primers **B,** AALV1 **C,** AoALV2 **D,** AoALV3 **E,** AoALV4 **F,** AoTV1; 100bp DNA Ladder (Thermo Fisher Scientific) was used in all analyses.

Table S1. List of primers used for direct virus RT-PCR detection

Virus	Direction	Sequences (5′-3′)	Tm (°C)	Product size (nt)
Armillaria ambi-like virus 1	Forward	CCCCTCATGGTCACTATGGATATG	61	800
	Reverse	CTCGCTGAGCCTCTCTACATCTT		
Armillaria ostoyae ambi-like virus 2	Forward	CGAGATGTTAGCCCTATACCCATC	60	487
	Reverse	GGTGTAGGAACTGTCTGTACTCTG		
Armillaria ostoyae ambi-like virus 3	Forward	CCGACTATAGGATTGTCCCTTCAG	60	477
	Reverse	CATCTGTCTTATCAGCTCTCTGGG		
Armillaria ostoyae ambi-like virus 4	Forward	CTATTCCCTTCCGACTTCTTTCCC	61	588
	Reverse	CTCTATCGAGACCAGATCCCAGATC		
Armillaria ostoyae tymovirus 1	Forward	GAGCTCTAGGTGAAGTGGAGTATG	60	731
	Reverse	CTCTCTTATAGGAACTCTAGGCGC		

Table S2. A, Pairwise Sequence Comparison (PASC) percentages (%) based on nucleotide sequence of ambi-like viruses hosted by *Armillaria cepistipes* and *ostoyae*. **B**, PASC % based on RdRP amino acid (aa) sequence of ambi-like viruses hosted by *Armillaria cepistipes* and *A. ostoyae*.

A

	AoALV1	AoALV2	AoALV3
**AoALV2**	27.66		
**AoALV3**	31.40	31.90	
**AoALV4**	85.73	29.82	30.09

B

	AoALV1	AoALV2	AoALV3
**AoALV2**	19.53		
**AoALV3**	77.46	18.65	
**AoALV4**	90.93	19.20	77.52

Table S3. Types and genomic position of the ribozymes detected in *Armillaria* ambiviruses.

Virus Name	Contig Name	Ribozyme (+)	e-Value	Ribozyme (-)	e-Value
**AoALV1**	ambi_spadesMETA_NODE_2_length_6862	HHRz (4510-4564)	1.4e-07	HHRz (330-254)	4.6e-11
**AoALV2**	ambi_spadesMETA_NODE_4_length_6697	HPRz (4424-4531)	5,00E-08	HPRz (4987-4884)	5.7e-10
**AoALV3**	ambi_spadesMETA_NODE_8_length_4562	HHRz (1727-1803)	7.7e-09	HHRz (2068-2014)	1.1e-08
**AoALV4**	ambi_spadesMETA_NODE_9_length_4549	HHRz (1-41)	0.021	HHRz (333-256)	2.5e-09

The (+) polarity is defined as the RNA strand coding for the polymerase (ORFA).
